# Assessment of Homonymous Recurrent Inhibition during Voluntary Contraction by Conditioning Nerve Stimulation

**DOI:** 10.1371/journal.pone.0167062

**Published:** 2016-11-23

**Authors:** Sidney Grosprêtre, Julien Duclay, Alain Martin

**Affiliations:** 1 INSERM CAPS UMR 1093, Cognition, Action and Sensorimotor Plasticity, Université de Bourgogne-Franche-Comté, Dijon, France; 2 EA4660, C3S Culture Sport Health Society, Université de Bourgogne-Franche-Comté, Besançon, France; 3 Toulouse NeuroImaging Center, Université de Toulouse, Inserm, UPS, Toulouse, France; University of Sydney, AUSTRALIA

## Abstract

In humans, the amount of spinal homonymous recurrent inhibition during voluntary contraction is usually assessed by using a peripheral nerve stimulation paradigm. This method consists of conditioning the maximal M-wave (SM stimulus) with prior reflex stimulation (S1), with 10 ms inter-stimulus interval (ISI). The decrease observed between unconditioned (S1 only) and conditioned (S1+SM) reflex size is then attributed to recurrent inhibition. However, during a voluntary contraction, a superimposed SM stimulation leads to a maximal M-wave followed by a voluntary (V) wave at similar latency than the H-reflex. This wave can therefore interfere with the conditioned H-reflex when two different stimulation intensities are used (S1 and SM), leading to misinterpretation of the data. The aim of the present study was to assess if conditioning V-wave response instead of H-reflex, by applying SM for both stimuli (test and conditioning), can be used as an index of recurrent inhibition. Conditioned and unconditioned responses of soleus and medial gastrocnemius muscles were recorded in twelve subjects at 25% and at 50% of maximal voluntary contraction at the usual ISI of 10 ms and an optimal inter-stimulus of 15 ms determined upon M- and V-wave latencies. Conditioned H-reflex (obtained with S1+SM paradigm) was significantly lower than the unconditioned by ~30% on average, meaning that the amount of inhibition was 70%. This amount of recurrent inhibition was significantly lower at higher force level with both methods. Regardless of the level of force or the conditioning ISI, results obtained with V-wave conditioning (SM+SM) were similar at both force levels, linearly correlated and proportional to those obtained with H conditioning. Then, V-wave conditioning appears to be a reliable index of homonymous recurrent inhibition during voluntary contraction.

## Introduction

During voluntary contraction, the motoneuronal output can be auto-regulated by a post-synaptic recurrent pathway involving the Renshaw cells. These cells receive among others a cholinergic motor axonal projection originating from motor neurons upon which they are projected [1–3]. The major roles ascribed to this recurrent pathway lie on the temporal (e.g. frequency, synchronisation) and spatial (synergist and antagonist distribution of the activities) control of the firing patterns of the motoneurons [4], acting as gain regulator of the motoneuronal output during voluntary contraction [5,6].

**Fig 1:**
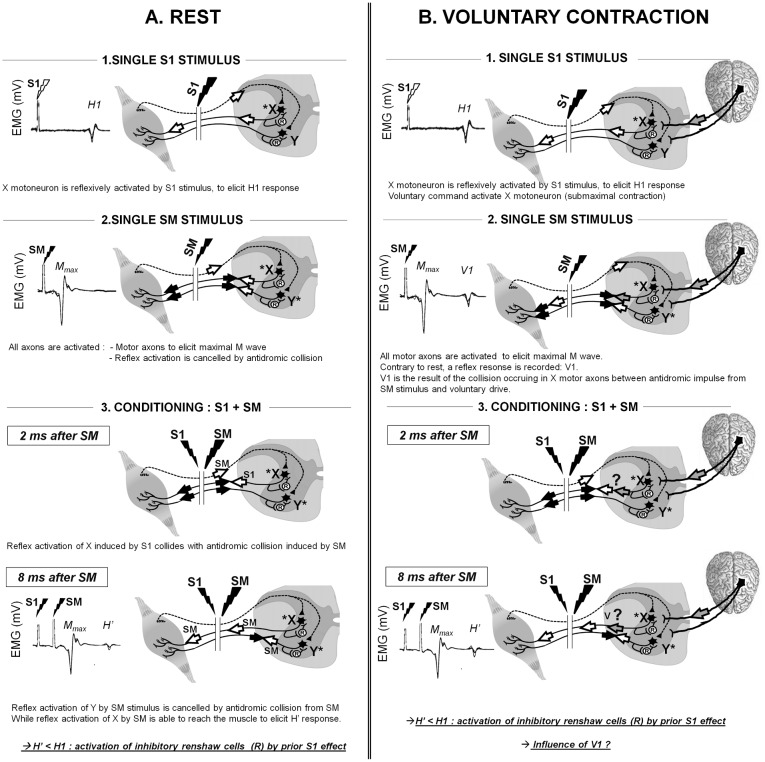
Illustration of the usual method to elicit conditioned H’ reflex response. The method consisting of evoking two stimuli over the peripheral nerve is described at rest (A) and during voluntary contraction (B). Each panel represents an EMG trace of the corresponding response (left) and a schema of the spinal circuitry (right) in which arrows represent the different volleys elicited directly on motor axons (black arrows), reflexively (white arrows) or by voluntary neural drive (grey arrows). Two alpha motoneurons are represented (X and Y) and are noted with an asterisk when they are activated. It can be noticed that both motoneurons activate a Renshaw inhibitory interneuron, noted R. A) 1. the response to single S1 stimulus is depicted: the stimulation of the peripheral nerve induced a depolarization of a certain proportion of Ia afferents and thus lead to activate motoneuron X to induce a single H1 reflex response. 2. the response to single SM stimulus is depicted: this stimulation will induce a depolarization of all afferents (Ia) and efferent fibres (motor axons). On one hand, the direct activation of all motor axons towards the muscle leads to a maximal M-wave (M_max_). On the other hand, the antidromic volley towards the spinal cord will collide with reflexive activation of all motoneurons (X and Y). 3. Combined conditioning (S1) and test (SM) stimulation at 10 ms intervals. At 2 ms after SM, the H1 reflex discharge of X motoneuron (white arrow) collides with the antidromic impulse from SM in X axon. 8 ms after SM, both motoneurones (X and Y) are activated by the Ia afferent volley elicited by the SM test stimulus: a reflex response develops in both motoneurones X and Y. However, this response is blocked in motoneurone Y due to antidromic collision but not in motoneurone X in which the collision already occurred with H1 reflex response. If H’ < H1: activation of inhibitory renshaw cell by prior S1 effect. B) The example of a submaximal voluntary contraction is depicted. Thus, only X motoneurons is activated by descending command. 1. The production of H1 response is similar to rest. 2. However, single SM stimulus during voluntary contraction induced a reflexive response (V1) because the antidromic collision occurred in X axon with descending volley (grey arrow), allowing the reflexive response from SM to reach the muscle. 3. During voluntary contraction, the mechanisms inducing H’ is rather difficult to interpret because of a confounding effect of the several inputs to the motoneurons from S1, SM and the descending volley. Particularly, the influence of V1 over H’ is not elucidated.

The method commonly used in humans to assess homonymous recurrent inhibition consists of delivering two electrical stimuli at short interstimulus interval (ISI) over the mixed peripheral nerve [7–9]. Fig 1 depicts a summary of the method. The first stimulus (S1) provides a prior reflexive activation of the motoneuronal pool via Ia afferent depolarization while the second is evoked at maximal intensity (SM) and recruits all axons available in the nerve, i.e. eliciting a maximal M-wave, M_max_ (Fig 1.1 and 1.2). With an appropriate ISI, the antidromic volley generated by SM will collide with the S1 reflexive volley in motoneuronal axons. Thus, the afferent activation of motoneuronal pool by SM, normally cancelled by the motor axonal antidromic volley, is allowed to reach the muscle again in the axons for which the collision between SM and S1 has occurred (Fig 1.3). The decrease observed between unconditioned H-reflex (S1 only) and conditioned response (S1+SM) called H', was then mainly attributed to recurrent inhibition [7,8], despite the fact that other mechanisms such as after-hyper-polarization or Golgi inhibitory circuit mediated by Ib afferents can also be involved, at least until 10ms after the first stimulus [10].

To specifically investigate recurrent inhibition, Pierrot-Deseilligny et al. in 1976 [8] have suggested that stimulation parameters, i.e. ISI between S1 and SM as well as the intensity of S1, have to be carefully set to ensure that the collision between S1 and SM occurs in motor axons (i.e., between the stimulation site and spinal motoneurons) and that S1 activates mainly the Ia afferent fibres (without motor axons activation). However, since this first investigation, most of the studies assessed recurrent inhibition with an ISI at a constant interval of 10 ms [9–12], despite the fact that the optimal ISI to record a valid H’ response seems to depend on the body height [9].

The validity of this method is even more dependent on the stimulation intensity used to evoke S1 [13]. Initially, S1 is set to record the maximal H-reflex amplitude without accompanying M-wave, as it was suggested that the greater the amplitude of the test reflex, the greater the amount of recurrent inhibition observed [13]. However, the fact that SM and S1 stimulus intensities differ can lead to the recording of a conditioned H’ response that does not necessarily activate the same pool of motor units than the unconditioned H response, especially during voluntary contraction [14]. In fact, SM delivered during voluntary contraction can lead to the recording of a reflexive response, i.e. V-wave [15], linked to a collision between voluntary descending neural drive and antidromic volley from SM (Fig 1B.2). This response, noted V1 in the present manuscript [15], is used as a marker of descending neural drive [16]. When the conditioning (S1 + SM) is applied during voluntary contraction, the H' response corresponds to afferent activation of the α-motoneurons by SM for which the collision between SM and S1, but also SM and descending drive, has occurred (Fig 1B.3). Also to avoid the confounding effect of this latter collision, the V-wave amplitude was generally subtracted from the H' response. [11,17]. However, V-wave amplitude was shown to increase [18] and the amount of recurrent inhibition to decrease [11] as the level of voluntary contraction increases. This phenomenon can lead to a negative value of H’-V difference that is difficult to interpret as a physiological phenomenon, and makes the assessment of recurrent inhibition difficult for moderate to high levels of contraction. To bypass this problem, a conditioning maneuver using SM intensity for both conditioning and test stimuli could be of interest for studying recurrent inhibition during voluntary contraction. Indeed, the use of the same intensity for both stimulations ensures that the same amount of motor axons is activated, allowing a more direct analysis of recurrent inhibition level through conditioned V-wave during voluntary contraction.

The aim of this study was to assess whether the experimental approach using V-wave conditioning can be a valuable method to analyze recurrent inhibition during voluntary contraction, at two different force levels of the plantar flexor muscles. The use of several levels of force would be useful to identify and compare the evolution of conditioned responses with both methods, as it is well known that recurrent inhibition levels decrease when the force level increase [11]. We hypothesized that the inhibition level observed through conditioning maneuver will decrease as the force level increase although unconditioned V-wave increases with the force level [16,18]. If variations of conditioned V-wave are proportional to those observed with H-reflex technique according to both levels of force tested, V-wave conditioning could therefore represent a valuable method to assess recurrent inhibition.

## Materials and Methods

Experiments were performed on twelve young healthy subjects (3 females and 9 males, age: 23.00±2.70, height: 1.76±0.09 m, weight: 69.86±12.62 kg). None of them reported neurological or physical disorders. After being fully informed about the investigation and possible related risks and discomfort, they all gave written informed consent to participate in the study. The experimental protocol was approved by the regional ethics committee (CPPGE—Comité de Protection des Personnes de la region Grand-Est) and was carried out in agreement with legal requirements and international norms (latest version of the Declaration of Helsinki). All experiments were carried out in one single session of about 2 hours.

### Mechanical Recordings

Experiments were performed on the right leg in a sitting position using an isokinetic dynamometer (Biodex system 3, Shirley, NY), with hip and knee joints at 90° (0° = full extension) and ankle joint at 90° (i.e., angle between the leg and the sole of the foot). The ankle was firmly strapped to the dynamometer with the motor axis aligned with the external malleolus of the ankle. During all experiments, particular care was taken in monitoring the subjects’ posture during the test and avoiding head rotations to maintain constant cortico-vestibular influences on the excitability of the motor pool [19]. The trunk was stabilized by two crossover shoulder harnesses. The dynamometer enabled instantaneous recording of muscle torque. For sub-maximal contractions, a feedback of force signal was monitored in front of the subject. The mechanical signals were digitized on-line (sampling frequency 2 kHz) and stored for analysis in TIDA software (Heka Elektronik, Lambrecht/Pfalz, Germany).

### Electromyographic Activity

EMG activity was recorded from three muscles of the right leg (soleus, SOL; medial gastrocnemius, MG; tibialis anterior, TA) using a custom made amplifier working with Heka acquisition system (Heka Elektronik, Lambrecht/Pfalz, Germany). After shaving and dry-cleaning the skin with alcohol to keep low impedance (< 5 kΩ), EMG signals were recorded by using two silver-chloride surface electrodes (8mm diameter) placed with an interelectrode center-to-center distance of 2 cm. For the SOL, the electrodes were placed 2 cm below the insertions of the *gastrocnemii* over the Achille’s tendon; for the MG electrodes were placed over the mid belly of the muscle; and for the TA electrodes were positioned at 1/3 of the distance on the line between the fibula and the tip of the medial malleolus [20]. TA EMG activity was recorded throughout the experiment in order to analyze the co-activation level between the several conditions. The common reference electrode was placed in a central position on the same leg (between stimulation and recording sites). EMG signals were amplified with a bandwidth frequency ranging from 15 to 5 kHz (gain = 1000) then digitized on-line (sampling frequency: 5 kHz) and stored for analysis with Tida software (Heka Elektronik, Lambrecht/Pfalz, Germany).

### Electrical Stimulation

The posterior tibial nerve (PTN) was stimulated via single rectangular pulses (1-ms width) delivered by Digitimer stimulators (model DS7A, Hertfordshire, UK). Two stimulators were used to modulate separately the intensity of the two stimulations used for double pulse stimulations, both connected to the same cable by a homemade electronic housing. PTN stimulations were elicited with a self-adhesive cathode (8-mm diameter, Ag-AgCL) placed in the popliteal fossa and an anode (5 x 10 cm, Medicompex SA, Ecublens, Switzerland) placed over the patella. The monitoring of TA EMG activity during the experiment allowed to ensure that the common peroneal nerve was not activated. Optimal stimulation site was first located by a hand-held cathode ball electrode (0.5-cm diameter) in order to obtain the greatest H-reflex amplitudes for the lowest stimulation intensity in both SOL and MG muscles. Once determined, the stimulation electrode was firmly fixed to this site with straps.

### Experimental Design

**Fig 2:**
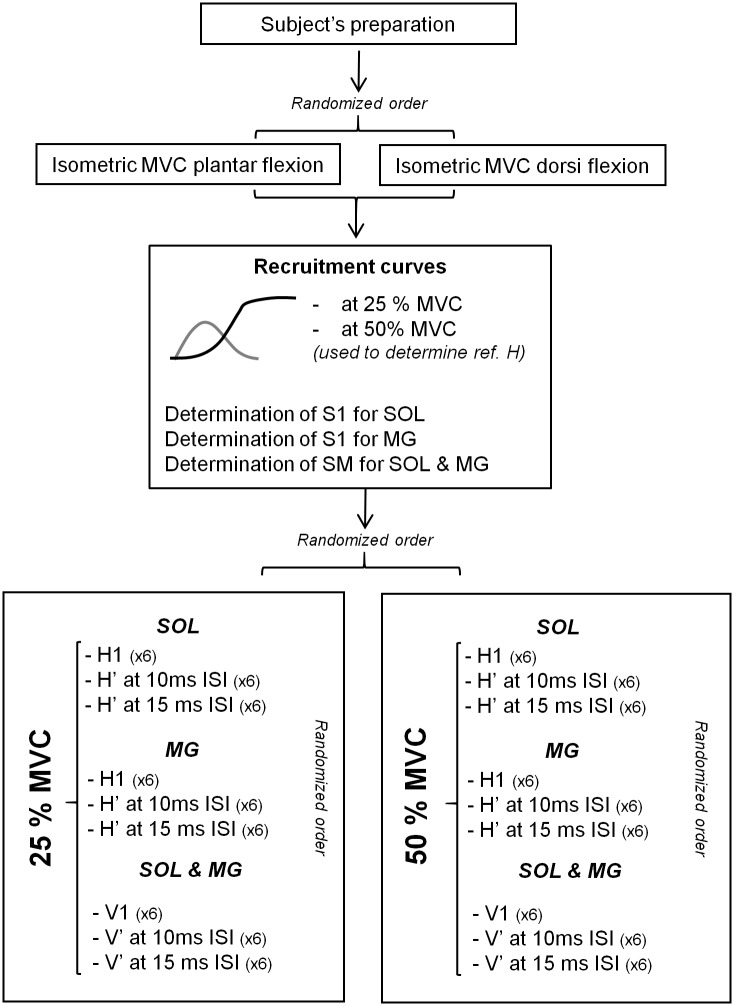
Experimental protocol. MVC: Maximal Voluntary Contraction. SOL: Soleus. MG: Medial Gastrocnemius. S1: stimulus intensity to provide H1 (unconditioned response). SM: stimulus intensity to provide M_max_ and unconditioned V-wave (V1). H’: conditioned H-reflex. V’: conditioned V-wave. ISI: Inter-stimulus Interval.

Experimental protocol is summarized in Fig 2. After subjects’ preparation, they were first asked to perform two isometric plantar flexion MVCs. If variations in maximal performance exceeded 5%, further trials were performed. The maximal value was then used to set the several levels of force required during the experiments. Levels of force assessed were 25 and 50% of plantar flexion MVC. To evaluate TA co-activation, maximal dorsi-flexion force was also evaluated with the same protocol. Then, to determine the optimal stimulation intensity needed to record H-reflexes and M-waves, the intensity of stimulation was increased from SOL and MG H-reflex threshold until M-wave no longer increased, with 2-mA increment. Four responses were evoked at each intensity, in order to build recruitment curves from SOL and MG muscles. Recruitment curves were established at 25% and at 50% MVC. The maximal intensity was then increased by 1.5 to record maximal M-waves (M_max_) to ensure that these waves, for both SOL and MG, lay in the plateau of their maximal value [21] (see Fig 2 for the full protocol). After the recording of the recruitment curves, two intensities were then determined and used in each muscle: the first to elicit maximal H-reflex with no associated M-wave called H1 [10] (S1) and the second to elicit M_max_ (SM). Contrary to SM, the S1 stimulation intensity used to evoke H1 was different between SOL (SOL S1) and MG (MG S1) muscles due to the difficulty to obtain such response at the same intensity for both muscles. To summarize, a total of 3 intensities were used: SM, SOL S1 and MG S1. Unconditioned H-reflex responses associated to S1 stimulus alone are noted H1. Similarly, unconditioned responses associated to SM stimulation only are noted M_max_ and V1 (V-wave accompanying M_max_). These responses were evoked when subjects performed voluntary contractions at 25% and at 50% MVC. Stimulations were automatically triggered and separated by constant intervals of 10 seconds. The force signal was monitored and subjects were asked to reach the plateau of 25% or 50% MVC by matching their mechanical signal to a line on the screen, 2 seconds before each electrical stimulation and to hold it for 2 s afterward. The evolution of the torque signal was carefully checked for every stimulations by the experimenter throughout the whole experiment. For each S1 stimulus (SOL S1 and MG S1), we ensured that the stimulus intensity provided H-reflexes without a visible M-wave. Thus, if necessary, stimulation intensity was re-adjusted.

Recurrent inhibition of SOL and MG muscles was then assessed by using double stimulations [7,9]. Prior to SM stimulus, conditioning stimulations were randomly delivered with the 3 different intensities (SM, SOL H1 and MG H1). Both conditioning intensities were tested under four randomly administrated conditions: two inter-stimulus intervals through two force levels (25 and 50% MVC). Six trials were recorded for each condition.

### Determining the Optimal Inter-Stimulus Interval (ISI)

**Fig 3:**
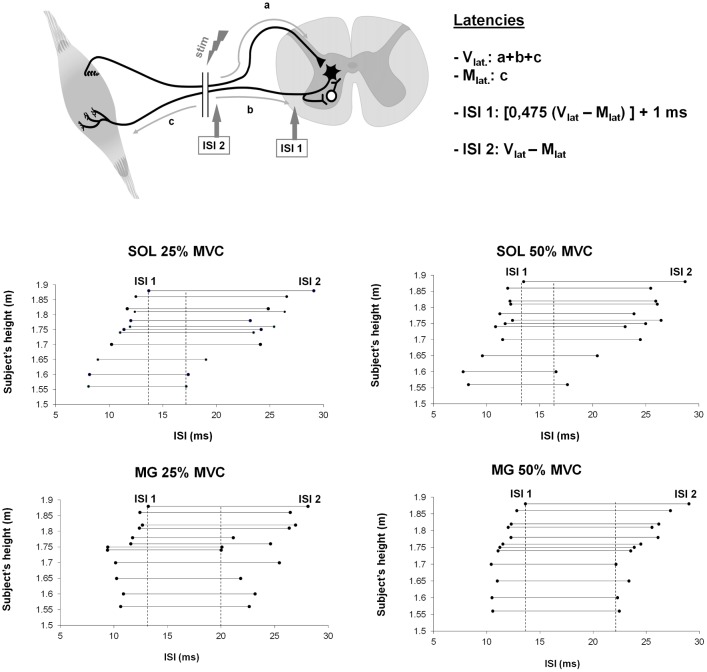
Calculation of the theoretical range of ISIs. In the upper panel the parameters of the formula used to determine appropriate theoretical interstimulus intervals (ISI) to record conditioned response are depicted. Maximal M-wave latency (M_lat_) and V-wave latency (V_lat_) were used to estimate these ranges in both SOL and MG muscles. ISI 1 represents the minimal ISI to record conditioned response, and ISI 2 the maximal one. In lower panels, ranges of theoretical ISIs for soleus muscle (SOL) and medial gastrocnemius (MG) are plotted against subjects’ height, for 25% MVC (left panel) and for 50% MVC (right panel). Vertical dotted lines indicate the common range of theoretical optimal ISIs for all subjects.

In the present study, the common ISI of 10ms [10] was used to ensure that in our conditions the results obtained with the H-reflex maneuver are in accordance with the literature. However, due to the limitations of using a fixed ISI without considering inter-subjects’ variability, particularly subjects’ height, we proposed a calculation allowing to define the range of ISI that allows the antidromic collision between stimulation to occur between stimulation site and spinal motoneurons (Fig 3). It is then possible to determine *a priori* a range of optimal ISIs by determining a range of theoretical ISI. Such type of calculation was already performed to assess the site where descending command and antidromic volley from nerve stimulation collide to lead to the recording of V-wave (see [16]).

Latencies of EMG responses were taken as the time interval from the onset of the stimulus artifact to the first peak of the responses (H, V, M_max_). Theoretical optimal ISI according to the subject’s height was determined from V1 and M responses latencies recorded in each condition (Fig 3), considering that H1 and V1 latencies were similar (SOL: F_1,11_ = 0.95, p = 0.35; MG: F_1,11_ = 1.71, p = 0.22). In order to increase the accuracy of the calculation, the differences between conduction velocities of afferent and efferent axons were taken into consideration in the formula. Indeed, it was previously shown that mean average conduction velocities of Ia afferents and alpha motoneurons were 50 m.s^-1^ and 45 m.s^-1^ respectively [22]. Thus, a ratio of 0.475 (average between afferent and efferent conducting velocity) was used for the determination of the duration of sections “a” and “b” depicted in Fig 3. This helped to determine the smallest optimal ISI (ISI 1, see Fig 3). A delay of 1 ms was added to provide enough time for the recurrent circuit to be involved, as this delay is commonly added to such calculation when one more synapse is involved [4,23]. The largest optimal ISI (ISI 2) was calculated by subtracting M-wave latency from V-wave latency. These calculations allowed providing a range of ISIs for which the antidromic volley from SM can collide with the reflexive volley induced by the conditioning stimulus in motor axons between spinal cord and stimulation site.

Based upon these results, 15 ms ISI was of particular interest since it was always included in the theoretical range of optimal ISIs whatever subject’s height or muscle tested in the studied population, contrary to the usual 10 ms ISI. Using a higher ISI of 15 ms than the usual 10 ms allowed to induce the desired collisions to record conditioned responses that actually reflect recurrent inhibition.

### Reference H-Reflex

Particular care was taken in recording the ascending part of H-reflex recruitment curve (i.e. from threshold of appearance until maximal H-reflex response), in order to provide unconditioned SOL and MG reference H-reflex. The reference H-reflex is an unconditioned response which amplitude matches the inhibited reflex amplitude by the conditioning maneuver. The reference H-reflex is used to provide an estimation of background excitability of motoneurons [11].

Reference H-reflexes analysis was made separately for SOL and MG as S1 intensity was different for both muscles. The reference H-reflex was taken among the unconditioned response taken from all stimulations evoked at the beginning of each experiment (recruitment curves, see Fig 2) that matches the amplitude of conditioned H-reflex at 25% MVC and 15 ms ISI. Reference H-reflexes were normalized by M_max_ of the corresponding condition. Then, to analyze excitability changes between conditions, the reference H-reflex was compared to the unconditioned H-reflex obtained at similar stimulation intensity during 50% MVC. This ensured that the modulation of conditioned H’ reflex from 25% to 50% MVC was not simply due to a change in background excitability.

### Data Analysis

The root mean square (RMS) values of SOL, MG and TA muscles EMG signals were determined with an integration time of 500 ms over the plateau of both levels of force, i.e. 25 and 50% MVC, prior the first stimulus. SOL and MG RMS were normalized by the corresponding M_max_. Then, to evaluate the TA co-activation level throughout the experiment, TA RMS during the several conditions was expressed as a percentage of TA RMS during maximal dorsi-flexion.

Peak-to-peak amplitudes of electromyographic responses were measured for quantitative analysis. Conditioned and unconditioned responses were normalized by M_max_ amplitude of each condition. It can be argued that analyzing normalized response (by maximal M wave of the same condition) is more suitable to compare several conditions that can affect spinal excitability [24]. Indeed, the increase in raw responses can be attributed to a rightward shift of the recruitment curve and not necessarily to changes in spinal excitability. In the present study only normalized responses have been considered, since conditioning effect was similar between raw and normalized responses.

As it is a commonly used tool to analyze conditioning effect, we also subtracted V1 amplitude from H’ responses in order to obtain [H’-V1] for each condition (level of force and ISI) and muscle (SOL and MG). The conditioned responses were expressed as a percentage of unconditioned to give the level of recurrent inhibition, using the following calculation: [(H’/H1)-1] x100 and [(V’/V1)-1] x100, for recurrent inhibition level obtained on H1 and V1 respectively.

### Statistical Analysis

All data are presented as the mean ± standard deviation. The normality of the data was tested using the Shapiro-Wilks test.

Latencies of unconditioned H-reflex responses were compared through a repeated measures ANOVA with factor *“level of force”* (25% and 50% MVC) and *“stimulus intensity”* (H1, V1) for SOL and MG muscles. For each muscle, separate three-ways repeated measures ANOVA were performed on normalized responses (H/M_max_ or V/M_max_) to assess the effects of factors “*inter stimuli interval*” (no conditioning, 10 and 15 ms), *“conditioning intensity”* (S1 and SM) and *“level of force”* (25% and 50% MVC). The same ANOVA was performed on percentages of inhibition.

Reference H-reflexes were compared to H’ by using a two-way repeated measures ANOVA with factors *“level of force”* (25% and 50% MVC) and *“conditioning”* (reference H, H’) on normalized responses (H/M ratios).

Main effects or interactions were followed-up by HSD Tukey’s tests. Statistical analysis was performed using STATISTICA (8.0 version, Statsoft, Tulsa, Oklahoma, USA). The level of significance was set at P < 0.05. The size effect was calculated by the partial eta-squared method as recommended by Levine and Hullett in 2002 [25]. Pearson correlations were assessed (percentages of inhibition induced by S1 *vs*. SM conditioning intensity), with P obtained in the Bravais-Pearson table (degree of freedom = 10). Coefficients of variation were determined as the ratio of standard deviation over the mean.

## Results

### Background Excitability

First of all, the TA co-activation levels did not show any significant differences between conditions (P = 0.81) and were on average 4.01 ± 0.64% at 25% MVC and 4.38 ± 0.98% at 50% MVC. As expected, EMG activities (RMS/M_max_) of the other tested muscles (SOL and MG) were significantly greater at 50% MVC than at 25% MVC (Table 1).

**Table 1:** Unconditioned responses.

	25% MVC	50% MVC
**SOL**		
RMS/M_max_ (a.u.)	0.066 ± 0,02	0.072 ± 0,01 *
Reference H (mV)	0.65± 0.26	1.04 ± 0.47 *
Reference H / M_max_	0.11 ± 0.07	0.09 ± 0.03
H1 (mV)	3.63 ± 0.82	4.46 ± 0.98 *
H1/M_max_	0.38 ± 0.05	0.40 ± 0.05
H1 latency (ms)	34.41 ± 1.6	34.93 ± 1.5
M_max_ (mV)	9.22 ± 1.05	10.52 ± 1.1
M latency (ms)	10.97 ± 0.5	11.11 ± 0.6
V1 (mV)	0.77 ± 0.15	1.94 ± 0.23 ***
V1/M_max_	0.10 ± 0.03	0.20 ± 0.06 **
V1 latency (ms)	34.36 ± 1.5	34.73 ± 1.5
**MG**		
RMS/M_max_ (a.u.)	0,058 ± 0,03	0,080 ± 0,04 *
Reference H (mV)	0.49 ± 0.16	0.64 ± 0.15 *
Reference H / M_max_	0.06 ± 0.02	0.08 ± 0.02
H1 (mV)	2.01 ± 0.32	2.57 ± 0.52 *
H1/M_max_	0.22 ± 0.04	0.26 ± 0.04
H1 latency (ms)	33.02 ± 0.9	34.06 ± 0.7
M_max_ (mV)	9.18 ± 0.97	9.67 ± 0.91
M latency (ms)	8.84 ± 0.31	9.22 ± 0.18
V1 (mV)	0.44 ± 0.07	1.23 ± 0.19 ***
V1/M_max_	0.05 ± 0.01	0.14 ± 0.04 **
V1 latency (ms)	32.96 ± 0.9	33.85 ± 0.8

*, **, ***: significantly different from 25% MVC, respectively at P<0.05. P<0.01 and P<0.001.

Regarding reference H-reflex, an interaction effect was found between factors l*evel of force* and *Conditioning* for normalized responses (SOL: F_1,11_ = 10.69, P = 0.007, ηP^2^ = 0.49; MG: F_1,11_ = 7.89, P = 0.017, ηP^2^ = 0.41). As expected, at 25% MVC normalized reference H-reflex was not statistically different from conditioned H’/M_max_ (P = 0.99 and P = 0.97 for SOL and MG respectively). However, H’/M_max_ was statistically greater than reference H/M_max_ at 50% MVC (P = 0.006 and P = 0.04 for SOL and MG respectively). Then, no statistical differences were found between normalized reference H-reflex at 25% MVC and at 50% MVC, for both muscles (Table 1).

### Unconditioned Responses

Latencies of V1 and H1 did not show any statistical difference (SOL: F_1,11_ = 0.95, P = 0.35; MG: F_1,11_ = 1.71, P = 0.22), and no effect of force level was found in either of the latencies (SOL: F_1,11_ = 1.34, P = 0.27; MG: F_1,11_ = 0.94, P = 0.35, Table 1).

Then, both SOL and MG H1/M_max_ did not show any significant difference with the factor *“level of force”* (SOL: F_2,22_ = 1.08, P = 0.36; MG: F_2,22_ = 0.93, p = 0.40). On the contrary, V1/M_max_ was significantly greater at 50% than at 25% MVC for both SOL (F_1,11_ = 22.11, p<0.001, ηP^2^ = 0.67) and MG (F_1,11_ = 23.86, p<0.001, ηP^2^ = 0.68).

### Conditioned Responses and Inhibition Levels

**Fig 4:**
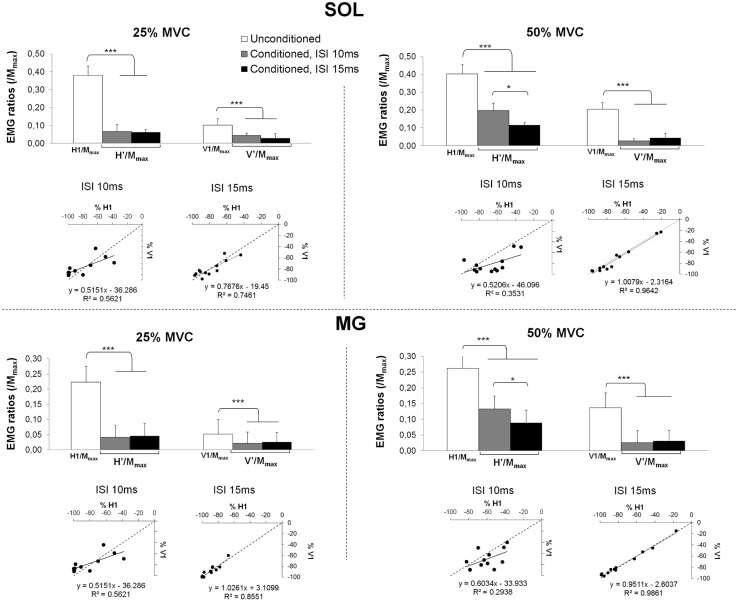
Conditioned and unconditioned responses of soleus (SOL) and medial gastrocnemius (MG) muscles. EMG ratios are depicted in upper panels. Unconditioned H1/M_max_ or V1/M_max_ (white bars), and conditioned responses H'/M_max_ and V’/M_max_ at 10 ms ISI (grey bars) and 15 ms ISI (black bars) are depicted for a conditioning stimulus set at S1 and at SM. In lower panels, percentages of inhibition (conditioned against unconditioned) induced by S1 (abscissa) are plotted against inhibition induced by SM conditioning (ordinate axis), for SOL and MG muscles at 25% and at 50% MVC. Dotted lines represent the identity line (x = y) and full lines represent regression lines of the relationships with their respective equations and coefficients (R^2^). *, ***: significant differences with unconditioned response respectively at P<0.05 and P<0.001.

An interaction effect was found on H/M_max_ and V/M_max_ between factors “*level of force*”, “*conditioning intensity*” and “*inter-stimuli interval*” for SOL (F_3,33_ = 7.64, P<0.001, ηP^2^ = 0.41) and MG (F_3,33_ = 3.38, P = 0.029, ηP^2^ = 0.35). Post-hoc analysis then revealed that regardless of the condition (ISI, conditioning intensity and level of force), a significant decrease was found between conditioned (H'/M_max_ and V'/M_max_) and unconditioned responses (respectively H1/M_max_, V1/M_max_; Fig 4). Conditioned H'/M_max_ or V’/M_max_ were greater at 50% MVC than at 25% MVC for both muscles at all ISIs (SOL: P<0.001; MG: P<0.001).

Regarding percentages of recurrent inhibition, a significant decrease was found from 25% to 50% MVC for SOL (respectively -77.27 ± 2.87% and -70.69 ± 5.73%; F_1,10_ = 16.25, P = 0.002, η_P_^2^ = 0.64) and MG (-81.19 ± 6.87% and -65.60 ± 10.34%; F_1,11_ = 16.24, P = 0.002, ηP^2^ = 0.60), i.e. the recurrent inhibition observed was statistically lesser at 50% than at 25% MVC. However, no statistical effect of the factor *“conditioning intensity”* (S1 *vs*. SM) was found on percentages of H and V inhibition (SOL: F_1,10_ = 2.43, P = 0.15; MG: F_1,11_ = 2.17, P = 0.17), i.e. the inhibition observed was similar with both methods. The most significant correlation between inhibition induced by the S1 conditioning and the one induced by SM conditioning was found at 15 ms ISI for both levels of force, r^2^ coefficient ranging from 0.29 to 0.56 at 10 ms ISI and from 0.75 to 0.98 at 15 ms ISI (see Fig 4), with a regression line close to the identity line (-x = -y).

The classical method consisting of subtracting V-wave amplitude from H’ showed negative values close to zero in nearly all conditions (ISI and force level). A great variability (coefficient of variation from 0.66 to 7.61) was found in H’-V1 differences; for SOL between– 9.00 ± 3.69% M_max_ (50% MVC, ISI 15 ms) and -0.71 ± 5.41% (50% MVC, ISI 10 ms); for MG between– 4.73 ± 2.14% M_max_ (50% MVC, ISI 15 ms) and– 0.34 ± 3.38% M_max_ (50% MVC, ISI 10 ms).

**Fig 5:**
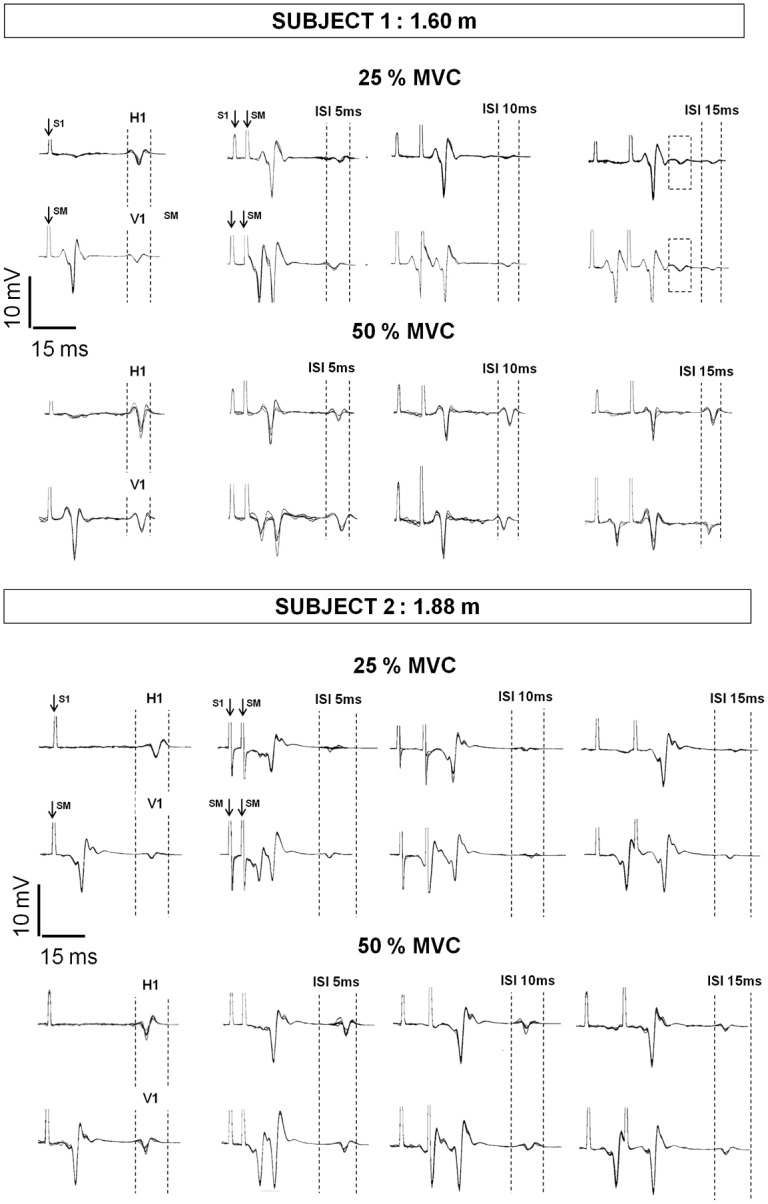
Representative results. Typical traces of SOL muscle in two representative subjects. Six traces for each condition are superimposed. The presence of two conditioned responses in subject 1 (e.g. at 15 ms ISI and 25% MVC) can be noticed, indicated by dashed squares.

Finally, it can be noticed that for some subjects (4 out of 12), a prior response to H’ or V’ appeared (Fig 5 subject 1). Interestingly, these subjects were the 4 smallest subjects of the group (height ≤ 1.75 m). The latency of this response (SOL: 34.96 ± 1.72 ms; MG: 33.52 ± 1.04 ms), measured from the onset of S1 stimulus artefact on EMG signal, was not different from the latency of H1 and V1. These responses were consistently observed before H’ and before V’ in the same four subjects and not in the other subjects.

## Discussion

This study aimed at investigating the double stimulation paradigm used to assess spinal recurrent inhibition in humans during voluntary contraction. Two methods were used and compared: H conditioning (common method) and V conditioning, at different levels of muscle contraction. The main finding was a similar inhibition induced in conditioned responses between both methods, whatever the muscle and the level of force tested.

As recurrent inhibition is one of the main post-synaptic mechanisms that modulate the output of alpha motoneurons, its evaluation is of particular interest during voluntary contraction [11]. Nevertheless, the method employed to assess recurrent inhibition can be rather complex to interpret. One of the main requirements to interpret conditioned H’ modulation is that the background excitability of the motoneuronal pool does not change between the several tested conditions, i.e. several force levels. The analysis of a reference H-reflex is commonly used as a tool to detect changes in background excitability which can account for different wave amplitudes between conditions [11,12]. When comparing reference H-reflexes normalized by maximal M waves of the corresponding condition, no differences were observed between the two force levels. It can also be noticed that previous studies that investigated recurrent inhibition still used raw amplitudes of conditioned and unconditioned reflexes in such analysis. In the present study, similar H1/M_max_ were also observed between both level of force, showing that the use of reference H-reflex might not be necessary when the analysis is performed from normalized ratios. Therefore, the fact that normalized responses were not increased from 25% MVC to 50% MVC emphasizes the assumption that the increase in raw responses can be attributed to a rightward shift of the recruitment curve and not necessarily to changes in spinal excitability background. According to previous methodological reviews and studies [21,24], the present study highlights the relevance of using normalized rather than raw responses.

Contrary to H1/M_max_ or reference H/M_max_, conditioned H’/M_max_ was statistically greater at 50% than at 25% MVC. These results, in line with those of Hultborn and Pierrot-Deseilligny [11], corroborated that recurrent inhibition level evolves with the level of force independently of the changes in background excitability of the motoneuronal pool. However, several other issues should be considered, as many mechanisms can be involved during voluntary contraction. Then, when using such conditioning method, a qualitative analysis of the recorded responses can also bring interesting clues about the underlying mechanisms. So, it can be noticed that the shape of the EMG signal recorded after conditioned stimulations differed from one subject to another, according to the ISI and force level (Fig 5). Indeed, it can be noticed that for some subjects a prior response to H’ or V’ appeared (Fig 5 subject 1). Given its latency, this earlier response observed before V’ or H’ represents the H-reflex response or the V-wave evoked by the conditioning stimulus. This response, which occurred with lower amplitude than conditioned response, may reflect an incomplete collision between the conditioning and the test stimuli. In fact, Bussel and Pierrot-Deseilligny [9] hypothesized that the optimal ISI depends on the size of the reflex pathway, which depends on the subject’s height. In a general way, 10 ms ISI was recommended as the shortest interval to use when assessing recurrent inhibition [10,12,26] to avoid the influence of other reflexive loops [10]. However, this interval may not be optimized for all subjects, especially for shorter subjects whose reflex pathway does not allow an effective collision between the two stimuli. In those subjects (n = 4/12 in the present study), two reflexive responses are recorded with conditioned stimulations whatever the method employed (H-reflex or V-wave conditioning) or the interval (10 or 15 ms). The fact that this second response was observed with H’ and with V’ in the same subjects, while the other subjects did not exhibit such response before H’ nor before V’, brings a first clue that similar phenomenon affected H-reflex and V-wave conditioning.

However, in these 4 subjects despite an incomplete collision a decrease is still observed in conditioned response compared to unconditioned, showing that other mechanisms than recurrent inhibition may be involved, such as after-hyper-polarisation mechanisms [27]. Others reflexive loops can also account for H-reflex depression by a conditioning stimulation, such as reciprocal inhibition mediated by antagonist Ia activation. In the present study, TA co-activation level during plantar flexion was also measured in each condition (ISI and force level). This level was constant throughout the experiment and we ensured, by monitoring TA EMG activity, that activation of antagonistic nerve was not activated. This showed that the impact of co-contraction mechanisms such as reciprocal inhibition was constant among the conditions and may therefore be ruled out from the present results. In addition, other inhibitory mechanisms, such as Golgi circuit, may not account for the present results since the tested ISI as well as the theoretical range of ISIs did not include short ISI (< 9 ms) [10]. However, during voluntary contraction, reflexive loops may not be the only mechanisms that modulated conditioned H-reflexes.

Contrary to rest, the descending neural drive onto the motoneuronal pool leads to the recording of a reflex response (V1) following maximal M wave. This response, which amplitude is commonly used as a marker to quantify the descending command, may widely influence recurrent inhibition. In fact, the link between V-wave response and conditioned H’ responses, both recorded during voluntary contraction, remains unclear. Hultborn and Pierrot-Deseilligny [11] suggested that V-wave response, which reflects the collision between voluntary command and antidromic impulses from the SM stimulus, can contaminate conditioned H’ response causing an over-estimation of its amplitude. Then, they proposed to subtract the amplitude of the V-wave (called V1 in the present study) obtained with single SM stimulus. Initially, Hultborn and Pierrot-Deseilligny suggested that only small V-waves can be subtracted from H’ responses (lower than 5% of M_max_), while another study subtracted the V-wave regardless the size [17]. Matching such V-wave amplitude may limit the assessment of recurrent inhibition to low levels of contraction. For instance, in the present study we observed that V1/M_max_ always exceeded 0.05 (Table 1), the limit fixed by Hultborn & Pierrot-Deseilligny [11]. Moreover, the amplitude of V1 was always greater than conditioned H’ response, leading to negative H’-V1 differences. The fact that different intensities, i.e. S1 and SM, are used to analyze recurrent inhibition is one of the main limits of this method. In fact, both stimuli do not induce similar responses according to the level of force. No effect of voluntary contraction level was found on H1/M_max_ (S1 stimulus), whereas a significant increase of V1/M_max_ (SM stimulus) was found from 25% to 50% MVC. We recently found that V-wave was well correlated to motor evoked potential amplitude elicited by transcranial magnetic stimulation and thus represents a reliable index of corticospinal neural drive [16]. Thus, comparing conditioning and conditioned V-waves would provide an interesting comparison between the amount of voluntary neural drive addressed to the motoneuronal pool and its recurrent regulation, since Hultborn and Pierrot-Deseilligny [11] suggested that recurrent inhibition was also dependent upon the amount of cortical neural drive. Indeed, in the present study, the so-observed increase in V-wave amplitude from 25% MVC to 50% MVC was associated with a decrease in recurrent inhibition, in accordance with the literature [10,11]. This was corroborated by a higher increase in H’ amplitude than in reference H-reflex from 25% to 50% MVC, emphasizing a lower recurrent inhibition level at higher level of force.

The high correlations observed between the inhibition levels induced by V-wave conditioning and those induced by H-reflex conditioning (Fig 4) suggested that the variations of conditioned responses are similar for both methods. This was emphasized by the fact that each relation was closed to the identity line (y = x), indicating that the decreases in V-wave are proportional to the decreases observed in H-reflexes. Thus, despite the fact that V1 and H1 showed different initial amplitudes, the conditioning maneuver induced similar inhibition. Regarding the usual H-reflex conditioning technique, it has been validated that during voluntary contraction the depression of conditioning response was of renshaw origin (e.g. [11,12]). The contribution of other mechanisms, such as after-hyper-polarization of alpha motoneurons, has already been ruled out by comparing several levels of force [9,28,29]. Animal experiments showed that the activation of descending pathways may reduce the level of after-hyper polarization [30,31] but this depression occurred 50 to 100 ms after the cortical activation of the alpha motoneurons [30]. This delay is incompatible with the present conditioning maneuver, regardless of the technique employed (H- or V-wave conditioning). Thus, according to these findings, as well as other supporting evidences from previous studies on V-wave origin (see [16]), V-wave is not subjected to such mechanisms and therefore can be affected by recurrent inhibition in a similar way as H-reflex during voluntary contraction. In addition, the decrease of recurrent inhibition showed in the present study between the low and the high level of force, which is consistent with the literature regarding H’ [11,12], was also observed by using V-wave conditioning maneuver, despite V-wave actually increased with the force level. The similar evolution of the conditioned responses from one force level to another, as well as the great correlations observed between both methods, emphasized the fact that V’/M_max_ can also reflect recurrent inhibition mechanisms.

To summarize, the present study showed that results obtained with V-wave conditioning, i.e. the use of SM intensity for both conditioning and conditioned stimuli, were consistent and well correlated with those obtained with the classical method. This consideration was particularly relevant when using the theoretically optimal ISI of 15 ms, calculated according to response latencies and subjects’ height. Thus, V-wave conditioning method, easy to implement with H-reflex measurement, could be of interest when studying transitional phenomena and allows assessing both recurrent inhibition and voluntary neural drive through V1 amplitude. In addition, this method allowed using the same stimulation for SOL and MG muscles since the M_max_ has reached a plateau in both muscles by the use of supra-maximal intensity. This helped recording conditioned response simultaneously in all triceps surae muscles, contrary to H1 recording which necessitated two different intensities between SOL and MG muscles.
